# Medical Items

**Published:** 1908-08

**Authors:** 


### MEDICAL ITEMS

---

ALBUMINURIA: The busy practitioner meets with many cases, in middle and advanced life, of scanty urination, with urine acidity, slight, or marked, albuminuria, and possibly some symptoms of cardiac or vascular disturbance. It is not possible, or expedient, in all these cases to diagnose Bright's disease, but, albuminuria with a falling off of the renal function is too suggestive of an approaching nephritis to justify any trifling in treatment. These cases call for the alkaline renal eliminants and a remedy like caffenio to act upon the circulation and induce diuresis. Alkalithia is such a combination and acts very promptly in the relief of such cases.

---

The tension on the sutures after an operation for epigastric hernia may be relieved by placing a pillow under the knees and propping the patient up in bed.

---

One should watch carefully for overdistention of the bladder in all cases of lesions of the spinal cord. In children the bladder has been known to distend sufficiently to hold 20-40 ounces.

Page(s) missing

